# Incorporating Single-Locus Tests into Haplotype Cladistic Analysis in Case-Control Studies

**DOI:** 10.1371/journal.pgen.0030046

**Published:** 2007-03-23

**Authors:** Jianfeng Liu, Chris Papasian, Hong-Wen Deng

**Affiliations:** 1 Department of Orthopedic Surgery, School of Medicine, University of Missouri-Kansas City, Kansas City, Missouri, United States of America; 2 Department of Basic Medical Science, School of Medicine, University of Missouri-Kansas City, Kansas City, Missouri, United States of America; 3 Laboratory of Molecular and Statistical Genetics, College of Life Sciences, Hunan Normal University, Changsha, Hunan, People's Republic of China; 4 The Key Laboratory of Biomedical Information Engineering of Ministry of Education and Institute of Molecular Genetics, School of Life Science and Technology, Xi'an Jiaotong University, Xi'an, People's Republic of China; University of Alabama at Birmingham, United States of America

## Abstract

In case-control studies, genetic associations for complex diseases may be probed either with single-locus tests or with haplotype-based tests. Although there are different views on the relative merits and preferences of the two test strategies, haplotype-based analyses are generally believed to be more powerful to detect genes with modest effects. However, a main drawback of haplotype-based association tests is the large number of distinct haplotypes, which increases the degrees of freedom for corresponding test statistics and thus reduces the statistical power. To decrease the degrees of freedom and enhance the efficiency and power of haplotype analysis, we propose an improved haplotype clustering method that is based on the haplotype cladistic analysis developed by Durrant et al. In our method, we attempt to combine the strengths of single-locus analysis and haplotype-based analysis into one single test framework. Novel in our method is that we develop a more informative haplotype similarity measurement by using *p-*values obtained from single-locus association tests to construct a measure of weight, which to some extent incorporates the information of disease outcomes. The weights are then used in computation of similarity measures to construct distance metrics between haplotype pairs in haplotype cladistic analysis. To assess our proposed new method, we performed simulation analyses to compare the relative performances of (1) conventional haplotype-based analysis using original haplotype, (2) single-locus allele-based analysis, (3) original haplotype cladistic analysis (CLADHC) by Durrant et al., and (4) our weighted haplotype cladistic analysis method, under different scenarios. Our weighted cladistic analysis method shows an increased statistical power and robustness, compared with the methods of haplotype cladistic analysis**,** single-locus test, and the traditional haplotype-based analyses. The real data analyses also show that our proposed method has practical significance in the human genetics field.

## Author Summary

Methods of haplotype-based analysis and single-locus analysis are widely used in genetic association studies. There is no consensus as to the best strategy for the performance of the two methods. Although haplotype-based analysis is a powerful tool, the large number of distinct haplotypes may reduce its efficiency. Haplotype clustering analysis is a promising way of decreasing haplotype dimensionality. A potential limitation of many existing clustering methods is that they do not allow the clustering to adapt to the position of the underlying trait locus. In this study, we proposed a weighted haplotype cladistic analysis method by incorporating a single-locus test into haplotype clustering. Under this framework, relationships between single loci and the disease outcomes can be considered when creating the hierarchical tree of haplotypes. The extensive simulations show that our method is robust against varied simulation conditions and is more powerful than either the original unweighted cladistic analysis method or single-locus analysis methods in case-control studies. Our hybrid method combining haplotype-based and single-locus analyses can be readily extended to whole genome association studies.

## Introduction

Recent advances in biotechnology such as high-throughput single nucleotide polymorphism (SNP) genotyping have provided useful tools to improve our understanding of the genetic basis of human complex diseases. With these advances, an intense and comprehensive evaluation of candidate genes, linkage regions, and the whole human genome can be conducted by genotyping dense SNPs.

Associations between genetic variants and disease outcomes are typically assessed using single-locus or haplotype-based analyses. Investigators have compared these two approaches to determine their relative efficiency in association studies, with somewhat inconsistent conclusions [1–9]. Some investigations believe that haplotype-based analysis provides higher power than single-locus tests [1–4,8,9]**,** while others have different opinions [6,7]. These different opinions may partially be attributable to different assumptions on SNP numbers and the linkage disequilibrium (LD) structure (particularly, frequencies and LD of markers and functional variants) at the locus of interest [10]. In general, haplotype-based approaches may have greater power than single-locus analysis when the SNPs are in strong LD with the risk locus [9]. In particular, haplotype-based analysis may be helpful in identifying rare causal variants [11].

Haplotype analysis is favorable for genetics association studies because it conserves joint LD structure and incorporates information from multiple adjacent SNP markers. However, as the number of SNPs within the region of interest increases, the number of distinct haplotypes increases rapidly. This may decrease the power and efficiency of the association tests by largely increased degrees of freedom (df) [12–19].

To tackle the problem of increased df in haplotype analysis, Templeton et al. [20] did their pioneer work using the haplotype cladistic analysis method. Since then, a series of haplotype-clustering methods was proposed for reducing the haplotype dimensionality in association studies. These methods can be broadly divided into two distinct categories. One is based on constructing tests based on comparing haplotype similarities between groups [21–26]. In haplotype similarity comparison method, the df of the test equals the number of markers studied within the haplotype region, which is usually much less than the number of distinct haplotypes. The other method aims at reducing the number of haplotypes by grouping distinct haplotypes into clusters and at comparing haplotype distributions based on clustered haplotypes rather than the original unclustered haplotypes [12,13,19,24,27–29].

In this study, we developed a novel haplotype-clustering approach that combines information from single-locus tests. Our method was developed based on the haplotype cladistic analysis method (CLADHC) originally proposed by Durrant et al. [19]. In our method, we combine single-locus tests and haplotype-based tests into a single test framework. Specifically, we incorporate information of single-locus tests into haplotype cladistic analysis by using *p*-values of single-locus test statistics to form weights that are used to construct distance metrics of haplotype pairs. By considering both single-locus and haplotype-based tests in haplotype cladistic analysis, we hypothesize that our method can improve the power and robustness of the association analysis. To validate our hypothesis, we generated the observed haplotypes by using Hudson's MS program [30], combined with similar simulation scheme of Durrant et al. [19]. We then conducted association studies under different scenarios for case-control designs. We compared the performance of our weighted cladistic analysis method with that of the CLADHC, single-locus allele-based test and the traditional haplotype-based analysis. The results show that our method is advantageous over the other three methods in terms of statistical power and robustness. Furthermore, we used the real data to compare the above four methods and found that our weighted cladistic method outperformed the other two haplotype-based analysis methods.

## Results

### Simulation Studies

From simulated 6-SNP haplotypes, we generated 24 sets of case-control samples using a complete combinatorial design based on the following parameters: three levels of heterozygote genotype relative risk (GRR) (1.5 and 1.75 versus 2.0), two types of genetic models (additive model versus dominant model), two levels of risk allele frequencies (0.1 versus 0.3), and two types of haplotype structures (high diversity versus low diversity). To evaluate the performance of detecting risk alleles based on our weighted cladistic analysis method, we conducted four association tests for each of the haplotype samples: (1) association tests based on the individual haplotype distribution without being clustered; (2) association tests based on the single-locus allele-based analysis; (3) association tests based on the clustered haplotype distributions obtained from CLADHC; and (4) association tests based on the clustered haplotype distributions generated from our weighted cladistic analysis method.

In our analyses, the log likelihood ratio (LLR) statistics under the logistic regression model are employed to test gene-disease associations using the four different methods aforementioned. In the analyses, we define the type-I error rate and the power as the proportions of significant associations reported in 2,000 independent replicates for the same marker under the null model (the GRR for the disease SNP was assumed to be 1.0) and the true disease model (the GRR > 1.0), respectively. Note that we report the significant associations for single-locus tests in terms of the maximal statistic for all the SNPs within the region considered.

We estimated the type-I error rates and powers of the four methods under different scenarios. In each scenario, we generated five sets of haplotypes with different dimensions (the number of distinct haplotypes varied between five and nine in the scenarios with low haplotype diversity, and between 11 and 15 in the scenarios with high haplotype diversity). Based on each set of haplotype within the same scenario, we performed 2,000 replication tests for disease-gene association to estimate type-I error rates and powers for each analysis method. The final results of the type-I error rate and power for each analysis method are averaged over the estimates obtained from the five sets of haplotype data within each scenario.

The type-I error rates of the association analyses for the four methods (at the 5% experiment-wise significance level) are presented in Table 1. All the methods, except the traditional haplotype-based method, are conservative to some extent due to Bonferroni correction for multiple tests, either between different partitions in both of the two haplotype clustering approaches or between different SNP loci in single-locus tests. The CLADHC procedure is the most conservative among the four analysis methods. Our weighted cladistic analysis method (denoted by “weighted” in Table 1) is less conservative compared with CLADHC and single-locus analysis. In contrast to the other three analysis methods that use Bonferroni correction, the traditional haplotype association analysis (denoted by “traditional” in the table) generated more reasonable estimates of type-I error rate. Both the haplotype structure and disease allele frequency (DAF) have no apparent influences on estimates of type-I error rate for each analysis method.

**Table 1:**
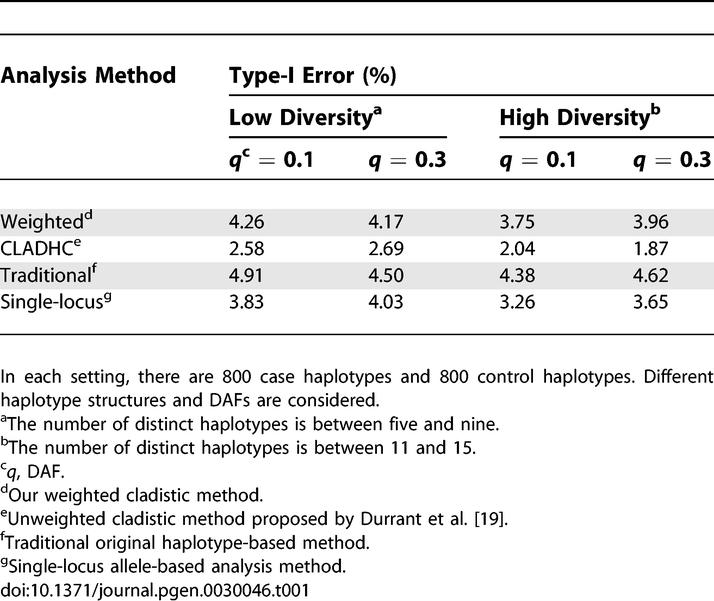
Type-I Error (%) of the Global Test (Accounting for Multiple Testing) Based on the LLR Statistic under a Logistic Regression Model at 5% Significance Level and Various Conditions

Table 2 shows the power for the four analytical methods to detect disease-marker association under the assumption of a 5% experiment-wise significance level, with Bonferroni correction for multiple testing in the two clustering methods as well as in single-locus allele-based analysis. The estimated power averaged over haplotype diversity is presented for each method under 24 different scenarios considering different DAFs, haplotype structures, heterozygote GRRs, and disease genetic models. Under each setting, we highlight the maximal power for emphasizing the best performance among the four analysis methods. It is within our expectation that the largest increases in power occur most frequently in our weighted clustering method.

**Table 2:**
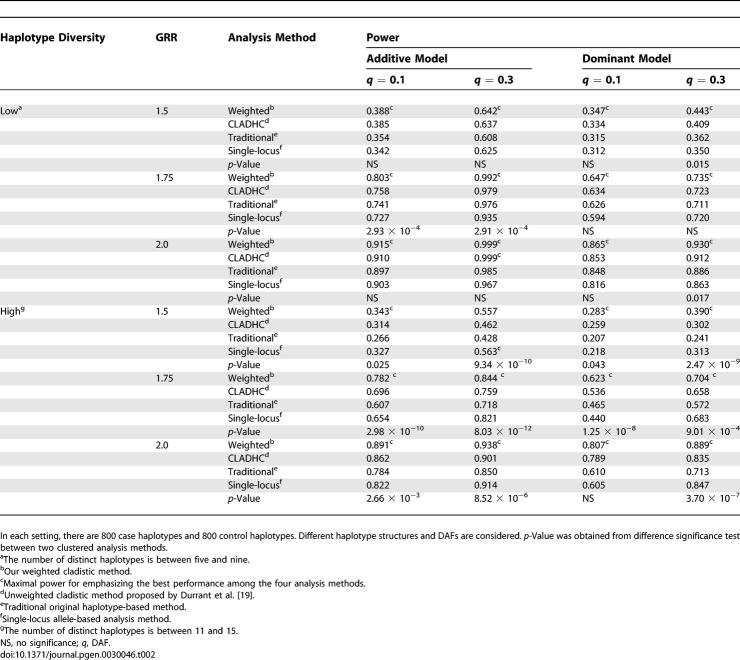
Powers of the Association Tests Based on the LLR Statistic under a Logistic Regression Model at 5% Significance Level

Comparison between two cladistic methods under different scenarios shows that the power of our weighted cladistic method is higher than that of CLADHC in the wide range of situations investigated. To formally test the difference between the two methods, we performed difference significance tests and obtained respective *p-*values under different scenarios presented in Table 2. Although the power of the two methods is comparable in some situations (nine of total 24 settings cannot reach significant level, i.e., *p*-value > 0.05), our method can substantially enhance the power in most simulated situations (15 of total 24 settings obtained the *p*-values < 0.05). This further confirms that, compared with CLADHC, our weighted cladistic method can enhance the power. An important point is that there was no power loss using our weighted cladistic method in all the simulations.

Comparison of the powers between the two clustering methods and with that of the traditional haplotype-based analysis method shows that clustering methods outperform the traditional method in all the simulated conditions. The power increase is more obvious for high diversity than for low diversity, and for small GRR than for high GRR. That is, when the original haplotypes have a higher dimensionality and the casual SNP entails a lower GRR, the two clustering methods have more advantages over the traditional haplotype-based method. These results suggest that reducing the df is of apparent benefit to power improvement in a trade-off against correction for the additional levels of multiple testing.

When comparing the performance across the three haplotype-based analysis methods and with that of the single-locus analysis method, our weighted cladistic method consistently shows advantages over the single-locus test in power level except by only one setting. However, the other two haplotype-based methods (CLADHC and the traditional haplotype-based analysis method) are not more powerful than the single-locus analysis method. Specifically, for the scenarios of low diversity haplotypes, power levels of CLADHC and the traditional haplotype-based analysis method exceed those of single-locus tests in most cases; however, for those scenarios of high diversity haplotypes, the single-locus analysis method shows better performance in most conditions than the two haplotype-based methods.

From Table 2, we can see that the highest power for all four methods is obtained under the combinational design of a higher DAF (0.3), a larger heterozygote GRR (2.0), a lower haplotype diversity, and the additive genetic model, and the power of all four methods is influenced by each of these parameters in a consistent manner.

**Figure 1:**
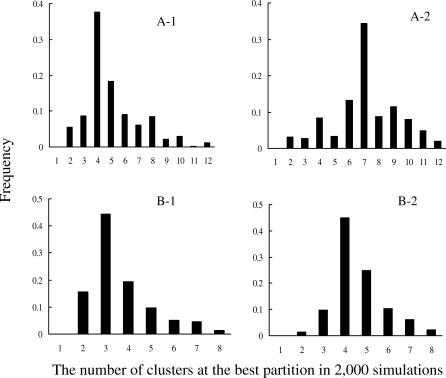
Examples of Distributions of Clusters at the Best Partition (T[best]) in 2,000 Simulations with the Two Clustering Methods under Different Settings A-1 and A-2 present the distributions of clusters at T[best] using our weighted cladistic analysis method and CLADHC, respectively, when original haplotypes have a high diversity (the number of distinct haplotypes is 12); B-1 and B-2 display the distributions of clusters at T[best] corresponding to using our proposed method and CLADHC when original haplotypes have a low diversity (the number of distinct haplotypes is eight). Other parameters considered in the simulations are the same for A-1, A-2, B-1, and B-2, which include: *q* = 0.1, *r* = 1.75, and assuming an additive model.

Finally, we investigated the distribution of the number of clustered haplotypes in the best partition T[best] (designated as the partition with the smallest *p-*value, i.e., maximal LLR value, among all separate LLR tests) in 2,000 simulations when using two clustering methods including our weighted cladistic method and CLADHC. Overall, under each different setting, the mode of this distribution in clustered haplotypes in T[best] ranges from three to six for haplotypes with low diversity and five to ten for haplotypes with high diversity in the two clustering methods. However, our proposed method has a smaller mode than CLADHC in most scenarios. This suggests that our weighted cladistic method tends to produce T[best] with fewer clusters compared to CLADHC. Thus, our weighted cladistic method may have a better performance to decrease the df of statistic than CLADHC in association analyses. Figure 1 presents examples of the distributions of the clustered haplotypes of T[best] in the two clustering methods.

### Real Data Analyses

To validate our proposed method, we applied it to analyze the published data by Gupta et al. [31]. In their studies, data from 120 unrelated rheumatoid arthritis (RA) disease individuals and 119 unrelated healthy individuals were collected to study the susceptibility of the mannose-binding lectin *(MBL2)* candidate gene. Haplotypes were defined by five intragenic SNPs of the *MBL2* gene, thus ten different haplotypes with frequencies >0.01 were observed. In the original analysis, one haplotype, CGCAG, was identified to show a significant difference in frequency between cases and controls (raw uncorrected *p-*value = 0.002).

In our analysis, we used four different methods including the original haplotype-based method, single-locus allele-based test, the CLADHC, and our weighted cladistic analysis method to perform association analyses between RA disease status and haplotypes of *MBL2* gene based on the data aforementioned. The *p-*value of the original haplotype-based analysis is 0.023, df being 9; the CLADHC used 3 df and has the *p-*value 3.90 × 10^−3^ (after Bonferroni correction); our weighted cladistic analysis method obtained the corrected *p-*value 3.97 × 10^−4^ using 2 df. Our method produced a *p-*value that is nearly 10-fold smaller than that of CLADHC and 60-fold smaller than that of original haplotype-based method. However, the *p-*value obtained from single-locus allele-based tests is 2.20 × 10^−4^ (after Bonferroni correction because of multiple loci), which shows no substantial difference from our method. The results suggest that our proposed method outperforms the other two haplotype-based analysis methods.

Table 3 presents the best partition of haplotypes of strongest association, together with the corresponding odds ratios for RA, when the cluster with the highest frequency of controls is taken as baseline. Cluster 3 has the highest odds of RA disease.

**Table 3:**
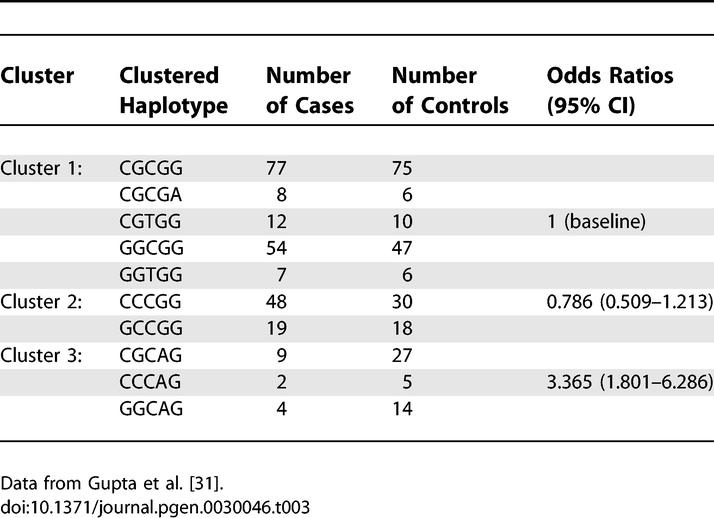
The Best Partitions of Haplotypes and Their Corresponding Odds Ratios for RA Disease

## Discussion

Haplotype analysis is likely to continue to play a key role in genetic epidemiology studies [32], because it effectively captures both the joint marker correlations and the evolutionary history. A main drawback of haplotype-based association tests is the comparatively large number of distinct haplotypes to be evaluated. As the number of haplotypes increases, the df for the corresponding test statistic also increases, thereby limiting the power of these tests.

Currently, the evolutionary-based clustering method is a useful tool to reduce the df in haplotype-based analysis. Some other clustering analysis methods were also proposed. For example, Seltman et al. [27] employed generalized linear models to analyze data for association studies. As an extension of the cladistic analysis method of Templeton et al. [20,33] and Templeton [20,33], their method is more flexible for its ability to deal with uncertainty of haplotype phases and allow for covariates. In Seltman et al. [27], the cladogram-collapsing algorithm was used to perform sequential statistical tests. The increasing size of cladogram nodes may lead to a very complex cladogram or network including many nodes each having only one or a few grouped haplotypes. Tzeng [13] also proposed a cladistic analysis method for association studies. The procedure of Tzeng [13] determines the cluster by preserving common haplotypes using a criterion built on the Shannon information content. Each haplotype is then assigned to its appropriate clusters probabilistically according to the cladistic relationship. An interesting feature of Tzeng's method is that the rare haplotypes can be grouped into the closest major haplotypes. This method requires phase-known haplotypes and does not handle covariates.

In addition to the aforementioned evolutionary-based clustering methods, Bayesian fine-mapping methods based on Markov chain Monte Carlo algorithm were also proposed, such as BLADE [34,35] and COLDMAP [36,37]. In BLADE, a Bayesian framework was developed using full haplotype information to handle various complications such as multiple founders, phase-unknown genotypes, and incomplete marker data. A stochastic model was employed to describe the dependence structure among several variables characterizing the observed haplotypes. A potential limitation is its assumption that the number of clusters is fixed by the analyst, which may not be robust if the number of clusters is misspecified [32]. The method of COLDMAP built many coalescent models for the genealogy underlying a sample of case chromosomes in the vicinity of a putative disease locus, which can incorporate the “shattered” coalescent model for genealogies and allows for multiple founding mutations at the disease locus and for sporadic cases. A major concern with these Bayesian fine-mapping methods is the computational burden due to Markov chain Monte Carlo algorithm, which may limit their applications in genome-wide scan studies.

It should be noted that a potential limitation of many existing clustering methods is that haplotype clustering is conducted without considering associations between haplotypes and the disease outcomes. That is, the clustering process does not use the information of phenotype data and the position of the underlying disease locus [32]. Given this consideration, we aim to develop a more informative haplotype similarity measurement. Here we propose a weighted cladistic analysis method, which incorporates information of single-locus tests into haplotype cladistic analysis, to perform association tests between disease phenotypes and clustered haplotypes. Our method is largely an improvement of Durrant et al. [19]. In the study of Durrant et al. [19], the authors used a simple form of the similarity metric to group the original haplotype, although they mentioned a general weighted form for calculating the similarity metric between pairs of haplotypes. Our method has several promising aspects. First, we construct a weighted distance metric for pairs of haplotypes through extracting the information from single-locus association analysis, and bridge a gap between single-locus analysis and haplotype-based analysis in case-control studies. Hence, we can group haplotypes based on both cladistic relationship of haplotypes and association between trait and SNPs within haplotype region of interest. Second, in CLADHC, haplotype diversity is assumed to be driven by marker mutation in the absence of recombination. In our weighted cladistic method, this assumption may be relaxed to some extent because the potential LD level between SNPs and disease gene can been partially captured by the constructed weight function −log(*p_i_*). We hypothesized that association tests combining the single-locus and haplotype methods are more favorable and powerful by incorporating their respective strengths into one framework of tests. In fact, extensive simulations showed that our method is robust and more powerful than either original CLAHDC or single-locus analysis in case-control studies.

Theoretically, our method may lead to inflations of type-I errors due to incorporating information from single-locus tests. This was confirmed in our simulation analyses by comparing estimates of type-I error rate between CLADHC and our method. However, the type-I error rate estimates in our method are still within the range of nominal significance level 5% in all 24 simulated scenarios. Since Bonferroni correction for multiple testing is conservative if the different test statistics are correlated, it may be more reasonable to determine the test thresholds using permutation procedure. Thus, to further confirm the gain in power of our weighted cladistic method, we performed tests by simulating null distributions of LLR statistics for the four different analytic methods based on permutation procedure, instead of using the theoretical null distribution of the statistic for traditional haplotype-based analysis method, or using adjusted *p*-values via Bonferroni correction for multiple testing for other three analysis methods. Because the permutational analogue is too time consuming, it is infeasible to analyze all sets of haplotype for the 24 scenarios we simulated. For illustration without losing generality, we only simulated one set of haplotype for each simulation scenario but kept the same simulation parameter of GRR = 1.5. For each simulated haplotype set, we performed 2,000 replications. In each replicate, the empirical critical values for different analysis methods were obtained by choosing the 95th percentile of the highest test statistic over the 1,000 permutation replicates. The results (unpublished data) demonstrated that when the critical values were obtained from permutation procedure rather than the theoretical null distribution and Bonferroni correction, our method still outperforms the other three methods, further validating gain in power of our weighted haplotype cladistic method. Although the results were obtained from a portion of the simulated haplotype sets, the overall trend of power increase has been clearly demonstrated. Therefore, the proposed method should be preferably acceptable for haplotype-based association studies for its robustness and the gain in statistical power.

In our simulation studies, we performed statistical tests based on phased haplotypes, which is not always available in practical studies. Commonly, we can infer haplotypes of phrase-unknown genotypes using the software HAPLOTYPER [38] or PHASE [39], which are widely used in the field. We can then conduct the subsequent analysis based on the inferred haplotypes. However, due to genotyping error and statistical haplotype reconstruction, phasing error or uncertainty of haplotypes is possible, especially for rare haplotypes. The rare haplotypes can increase df, resulting in a decrease of power in haplotype-based association tests. A common practice is to discard the rare haplotypes, which may result in information loss as current statistical methods cannot completely distinguish between the real rare haplotypes and rare haplotypes because of genotyping error. An alterative method is to pool the rare haplotypes into a single baseline group, this method is widely used in the field [40–42]. However, it may be difficult to interpret the odds ratio of the pooled rare haplotypes in association analyses, unless we assume all the rare haplotypes have the same genetic effect. An appealing approach summarized in Schaid [32] is to “shrink” the effects of rare haplotypes. The shrinkage can be either toward a common mean, with the effects of the rare haplotypes shrunk somewhat to the same degree as those haplotypes with which they are most similar, or toward the effects of the haplotypes that are most similar to the rare ones [32]. In our analyses, we pooled the clusters with relative sample frequencies <5%. We believe that the problem by pooling rare haplotypes here is not a serious issue in our study. The reason is that the hierarchical clustering technique is a natural way to cluster the rare haplotypes according to distance metric among haplotypes. In the clustering process, rare haplotypes were firstly grouped according to distance metric among haplotypes, and those rare clusters under the cut-off threshold (5%) were pooled. Thus the proportion of rare haplotypes being pooled in the best partition was virtually low among the 2,000 simulation replications (the proportion of pooled rare haplotype group was 0 in most cases under scenario of low diversity, and varied from 0% to ∼15% under a scenario of high diversity). In contrast, in traditional haplotype-based analyses, rare haplotypes under cut-off threshold were pooled directly and, accordingly, the proportion of pooled haplotypes varied from 0% to ∼37.5% (three out of eight) for scenario of low diversity, and from ∼13% (two out of 15) to ∼38.5% (five out of 13) for high diversity. In this study, we adopted 5% as the cut-off threshold for pooling the rare haplotypes, which is commonly used in the field. Lowering the threshold (e.g., from 5% to 2.5% or to 2%) may be helpful to keep the size of the pooled rare haplotype group under better control, as we can avoid pooling those “moderate” rare haplotypes each having quite different haplotypic effect under a lower threshold. This is a topic that we will pursue in future studies.

Here our simulations are largely based on phase-known data*.* For uncertainty of haplotypes inferred from phase-unknown data, if only the most likely haplotype configurations are used, it may cause a loss of information and potential bias in the subsequent analyses. As summarized in Schaid [32], we can adopt the following steps to handle the uncertainty of haplotypes: (1) enumerate the possible haplotypes by suitable haplotype reconstruction software; (2) reconstruct the hierarchical tree for those enumerated haplotypes using our weighted haplotype distance metric; (3) develop a design matrix, with the columns corresponding to haplotype clusters and the rows corresponding to all individuals. At the *i*th row of the matrix, for each possible pair of haplotypes carried by individual *i*, the columns can be used to count the clusters that individual *i* haplotypes are grouped into; (4) average design matrix row by row for each individual according to the posterior probabilities of those phase-unknown haplotypes; and (5) the averaged design matrix can be used in logistic regression model to perform the LLR test.

Recent advances in high-throughput genotyping technology have made it feasible to use empirical LD patterns to search the whole human genome for disease risk variants. The sliding windows approach combined with haplotype-based association represents one of the most suitable methods to perform whole genome association (WGA) studies. Several groups have explored this approach from both statistical [43,44] and applied perspectives [45–47]. Our proposed weighted cladistic method can be easily adapted for WGA studies using the sliding window approach. For example, our method can be used in WGA studies by the following procedures: (1) haplotype reconstruction (softwares are available, such as HAPLOTYPER [38] or PHASE [39]) and haplotype block partition (htSNPer [48] or HaploBlockFinder [49]) for whole genome genotype data; (2) in each haplotype block, reconstruct the hierarchical tree within each of the sliding windows using the weighted haplotype distance metric, and detect association between clustered haplotype and disease outcomes in each window; and (3) correct for two levels of multiple testing including the number of blocks and the number of windows in each block. It should be noted that the number and the length of sliding windows have obvious impacts on the results, because the long windows might include haplotypes with recombination, while many short windows increase the stringency to reach statistical significance due to the need to correct for multiple testing. Compared with CLADHC, a strength of our method is that the assumption of no recombination within each sliding window (which is not always held in practice) is not strictly required, because our method can partially capture information of recombination between markers and disease gene by the constructed weight function −log(*p_i_*). Therefore, longer sliding windows can be applied with no extra power loss when performing WGA using our weighted cladistic analysis method.

Optimizing the length of sliding windows is important for WGA studies. A commonly used method to optimize sliding window size is through identifying regions of high and low LD. Thus, the constructed windows can reflect different amount of LD in the data. Generally, we can adopt windows of large sizes for genomic regions of extensive LD, and small sizes for regions of moderate or weak LD. However, in practice, it is not always easy to obtain the optimal window sizes [50]. Another commonly used method is to use windows of variable sizes to screen regions densely genotyped. That is, for a given maximal window width, all possible widths of windows are utilized to find the strongest evidence of association (the maximum statistic) for each locus under investigation [51–53]. However, the issue of thousands of tests is a stumbling block for detecting the causal variant. Recently, Mathias et al. [54] proposed a new method named Graphical Assessment of Sliding *p*-values, which provides a graphical overview of all tests from sliding windows without subselection, and thus may alleviate the multiple testing problem to some extent.

In our single-locus analysis, we performed allele-based association tests at each SNP under logistic regression model. Analysis based on alleles regardless of the genotypes is counter-intuitive, which can provide the most powerful method of testing under the multiplicative genetic model [55]. Under this framework, the assumption of HWE is essential. If departure from HWE is seen for the genotype data, we can directly analyze genotype data per se instead of basing on allele counting method. In our study, for ease of comparison among different method, LLR tests under logistic regression model were used to detect gene-disease association in all four different analysis methods aforementioned. Compared to the conventional Pearson's χ^2^ test for contingency table, the logistic regression analysis can construct a better fitting and biologically more reasonable model to describe the relationship between disease status (dependent or response variable) and a set of independent variables including markers and covariates.

In summary, we report here a weighted haplotype cladistic method that is capable of effectively constructing a cladogram of distinct haplotypes by incorporating associations between single marker loci and phenotype data. Compared with the original CLAHDC, traditional haplotype-based analysis, and single-locus analysis methods, our proposed method can substantially improve the power of association tests and is more robust for a variety of simulation conditions for the case-control design.

## Materials and Methods

In our method, we determined haplotype diversity by the proportion of allele matches at each SNP locus within a haplotype region under a mutation model. In the mutation model, mutations at marker loci resulted in haplotype diversity, and no recombination events happened [19,25,28]. This is the same model as that used in the CLADHC. This metric of haplotype diversity will be used to construct cladograms of haplotypes using standard hierarchical clustering procedures [56]. If a haplotype covers the disease susceptible mutation, the cladogram can be approximately regarded as the genealogical tree underlying the shared ancestry of case and control haplotypes [19]. Therefore, association between disease and haplotype clusters in the cladogram can be detected because those clusters containing mutated haplotypes share more recent common ancestry than those containing nonmutated haplotypes.

We evaluated the proposed weighted cladistic analysis method by performing simulation studies using a case-control design and compared false positive error rates (type-I error rates), powers of our method with those of single-locus allele-based analysis, CLADHC, and traditional haplotype-based methods.

### Definition of distance metric.

Construction of distance metric between pairs of haplotypes in our method includes two steps: First, we performed single-locus allele-based analysis using an LLR test based on the logistic regression model under the case-control design. Second, we employed the *p-*values obtained in single-locus tests to calculate weights. These weights were assigned to the similarity index of haplotype pairs at each corresponding SNP locus. We used the weighted similarity to define the distance metric between pairs of haplotypes.

We considered *n* tightly linked SNPs in a region of interest. We assumed that haplotype phase information is known. The pair of haplotypes carried by individual *k* is denoted by H*_k_* = {H*_k_*_1_,H*_k_*_2_}, and the haplotype H*_kj_* = {H*_kj_*_[1]_,H*_kj_*_[2]_,…,H*_kj_*_[*n*]_} (*j* = 1, 2). We coded two different alleles at SNP *i*, H*_kj_*_[*i*]_, 1 and 2 (code 2 denotes the minor allele), respectively. The frequency of allele 2 at SNP *i* is *q_i_*.

We assumed that there were *m* distinct haplotypes for a chromosome region carried by a sample of unrelated cases (affected) and controls (unaffected). Following the basic idea of CLADHC [19], we employed a cladogram to depict haplotype diversity for these *m* distinct haplotypes, which can be depicted by a similar figure example elsewhere (referring to Figure 2 in reference [19]). At the bottom of the cladogram, *m* distinct haplotypes are treated as *m* clusters in the first partition, T[*m*]. At the top of the cladogram, all distinct haplotypes are merged in a single cluster in the last partition, T[1]. From partition T[*m*] to T[1], all successive merging are formed stepwise according to the distances between clusters.

We constructed cladograms using simple hierarchical group averaging techniques. At each partition, clusters of haplotypes with a minimum average distance from the previous partition are merged, and thus the mean pairwise haplotype diversity is minimized within the new clade. We constructed the distance metric to represent the diversity between a pair of haplotypes, *H*_*k*_1_*j*_1__ and *H*_*k*_2_*j*_2__:

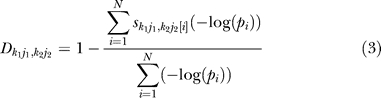
where −log(*p_i_*) acts as the weight assigned to the similarity,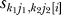 , at locus *i*, and *p_i_* is the *p-*value obtained in single-locus allele-based association analysis at SNP *i* using traditional Pearson's χ^2^ test. The similarity of two haplotypes at SNP *i*,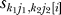 , can be given by:

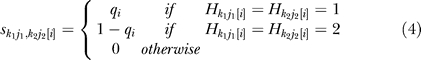



As shown in Equations 3 and 4, haplotypes that share rare alleles are believed to share more recent ancestry than haplotypes sharing common alleles and thus show greater similarity by means of the definition of haplotype diversity. Therefore, the complementary allele frequency, i.e., *q_i_* for sharing allele 1, and 1−*q_i_* for sharing allele 2 at SNP *i*, is used to evaluate allele sharing. Furthermore, −log(*p_i_*) is treated as the weight to the similarity at locus *i*, which means that a SNP with a lower *p-*value in single-locus analysis will play a more important role in determining the distances between haplotypes. To some extent, a lower *p-*value reflects stronger evidence of LD between the marker and the putative disease mutation. Therefore, if a lower *p-*value is obtained at a SNP locus, the pair of haplotypes sharing alleles at this locus will have a higher probability of sharing alleles of the disease mutation. Correspondingly, the pair of haplotypes with mismatched alleles at this locus will have a lower probability of sharing alleles of the disease mutation.

We use our weight-based distance metric to successively merge original distinct haplotypes into different clusters in the hierarchical cluster framework, and thus original distinct haplotypes within a cluster can be regarded as the same haplotype in the next round of merging. Therefore, association analysis between clustered haplotypes and disease phenotype can be conducted in case-control studies.

### Statistical tests.

A weighted cladistic analysis using LLR test statistic under a logistic model was used. After reconstruction of the hierarchical tree using our weighted haplotype distance matrix, we performed association analysis between clustered haplotypes and disease at each partition included in the cladogram based on the logistic regression model, which is essentially the same as that of Durrant et al. [19]. A general description of this statistical test method is provided in Protocol S1.

The traditional haplotype-based analysis method used in our study refers to the method that directly analyzes haplotype data based on LLR test under logistic regression model. In the analysis, we treat each haplotype with a frequency ≥5% as a distinguishable “cluster” and pool those haplotypes with relative frequencies <5% into a single baseline group. As such, the LLR test statistic with df *m*−*l* is used to perform haplotype/disease association. Here *m* and *l* denote the numbers of distinct haplotypes and those haplotypes being pooled, respectively. Equations A1–A4 in Protocol S1 can be adopted in traditional haplotype-based analysis by changing independent variables 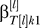 and 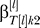 in Equation A1 to *β_k_*_1_ and *β_k_*_2_, respectively. Here, *β_k_*_1_ and *β_k_*_2_ denote the log-odds of two haplotypes H*_k_*_1_ and H*_k_*_2_ carried by individual *k*. Similarly, we denote *β_k(pool)_* as the log-odd of either haplotype with a relative frequency <5% carried by individual *k*.

Single-locus allele-based analysis was also performed by using LLR test statistic under a logistic regression model. Comparisons among the four different analysis methods are based on the same framework. The LLR statistic construed at each SNP locus within the haplotype region follows a χ^2^ distribution with 1 df under the null hypothesis that cases and controls have equal odds of carrying each allele. The models used for traditional haplotype-based analysis can be employed here to test SNP-disease association by treating *β_k_*_1_ and *β_k_*_2_ to be the log-odds of two alleles at each locus instead of two haplotypes carried by individual *k*. The raw *p*-value obtained from each single-locus test is used to form weight for constructing distance metric between haplotype pairs in the subsequential weighted cladistic analysis. The minimal *p*-value among all separate tests is adjusted for multiple testing with Bonferroni correction and then is regarded as the evidence of association.

To confirm the gain in power of our weighted cladistic method compared to CLAHDC, we constructed a test statistic to formally test the difference of power between the two methods.

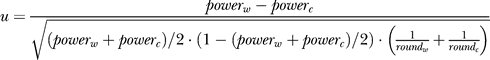
where, *power_w_* and *power_c_* are the power estimates for our method and CLADHC, respectively, and *round_w_* and *round_c_* are the simulation replicates in power estimation. The test statistic *u* approximately follows a stand normal distribution under null hypothesis of no difference in power between the two methods.

### Simulation scheme.

In our study, we generated SNP haplotypes and disease phenotypes by three steps. First, we used the MS program developed by Hudson [30], which mimics haplotype data based on the coalescent theory to simulate haplotypes. Second, a certain SNP is designated to be the causal variant of a complex disease, which is used to determine disease status. Third, the causal variant is removed from the original simulated haplotype. In this case, we perform disease-gene association under an “indirect” association framework (that is depending on LD between the markers and the causal variant), which is quite similar to the simulation scheme of Durrant et al. [19].

We set the main parameters under the coalescent model for generating haplotype data as follows: (1) the effective diploid population size *n*_e_ being 1 × 10^4^; (2) the scaled recombination rate for the whole region of interest, *ρ* = 4*n*_e_ *γ*/bp, set to be 4 × 10^−3^ and where parameter *γ* is the probability of cross-over per generation between the ends of the haplotype locus being simulated; (3) the scaled mutation rate for the simulated haplotype region, *θ* = 4*n*_e_ *μ*/bp, set to be 8 × 10^−4^, and where parameter *μ* is the neutral mutation rate for the region of simulated haplotypes; and (4) the length of sequence within the region of simulated haplotypes, *n* sites, being 10 kb. These parameter values are often used in earlier analyses [13,30].

Based on these parameter settings, we ran the MS program to generate the SNP sequences of the haplotype sample and set the number of SNP sequences in the simulated sample at 100. We discarded rare SNPs with minor allele frequencies lower than 0.05. We also defined a haplotype as a segment including seven contiguous SNPs within the simulated SNP sequence region, where we fixed the fourth SNP as the liability locus affecting a complex disease. Liability alleles were determined according to DAF *q* (*q* = 0.1 and 0.3). We considered two types of haplotypes with different structures within the region of simulated sequences in our studies, i.e., haplotypes with low diversity (the number of distinct haplotypes ranges between five and nine) and those with high diversity (the number of distinct haplotypes ranges between 11 and 15).

With the assumption of a single liability allele with a moderate effect underlying a complex disease, we generated samples of cases and controls based on the following settings. Denote *f_i_* as the penetrance function, which is the probability of being affected conditionally by carrying *i* copies of the risk allele (*i =* 0, 1, or 2). We defined the ratio of *f_1_*/*f_0_* as heterozygote GRR and set the disease prevalence *K* = 0.01. We let *r* = *f_1_*/*f_0_*. Given parameters *r, K,* and *q*, we obtained *f_0_* = *K*/(1 − 2*q* + 2*qr*). Then we obtained *f*_1_ and *f*_2_ under different genetic models. When an additive model was considered, we had *f*_1_ = *rf*_0_ and *f*_2_ = 2*rf*_0_ − *f*_0_; if a dominant model was considered, we had *f*_1_ = *rf*_0_ and *f*_2_ = *f*_1_. After determining the values of *f*_0_, *f*_1_, and *f*_2_, we randomly drew two haplotypes from the simulated sample containing 100 7-SNP haplotypes and paired them to form an individual. Thus the probability of the individual being a case was *f_i_,* which was only determined by *i,* the number of copies of risk alleles at the liability locus. We repeated this process till *n*/2 cases and *n*/2 controls were formed. In our study, *n* = 800. Finally, we removed the fourth SNP from simulated 7-SNP haplotypes to form “observed” 6-SNP haplotypes for all case and control individuals. These 6-SNP haplotypes were used to conduct disease-gene association analysis in the simulation studies.

### Program availability.

We employed SAS e9.1 to code our proposed method in the Windows XP environment. The program is available upon request.

## Supporting Information

Protocol S1 LLR Test Method under the Logistic Regression Model for Detecting Association between Grouped Haplotypes and Disease Phenotypes (36 KB DOC)

## Abbreviations

CLADHC: haplotype cladistic analysis method
DAF: disease allele frequency
df: degrees of freedom
GRR: genotype relative risk
LD: linkage disequilibrium
LLR: log likelihood ratio
RA: rheumatoid arthritis
SNP: single nucleotide polymorphism
WGA: whole genome association
