# Supplementary material for: Incorporating Single-Locus Tests into Haplotype Cladistic Analysis in Case-Control Studies
Source: PLoS Genet. 2007 Mar 23;3(3):e46. doi: 10.1371/journal.pgen.0030046 (PMC1829402; doi:10.1371/journal.pgen.0030046)
Supplement: Supplementary file 1 [file pgen.0030046.sd001.doc]

**Protocol S1**

The *LLR* test method under the logistic regression model for detecting association between grouped haplotypes and disease phenotypes

Among successive partitions from T[*m*] to T[1], consider a partition of haplotypes, T[*l*], with *l* clusters. The disease status of individual *k* can be denoted by *y_k_* = 1 for a case and *y_k_* = 0 for a control. In a logistic regression framework, we denote *log-odds* of disease for *l* clusters by$\text{β}^{[l]}=\{\beta_{1}^{[l]},\beta_{2}^{[l]},...,\beta_{l}^{[l]}\}$, and covariates, denoted by **x***_k_* **= {***x_k1_*,*x_k2_*,…,*x_kC_***}** for individual *k***,** with corresponding regression coefficients $\phi^{[l]}=\{\phi_{1}^{[l]},\phi_{2}^{[l]},...,\phi_{C}^{[l]}\}$, which can also be allowed to enter the model. We also denote the *log-odds* of two clusters at partition T[*l*] by $\beta_{T[l]k1}^{[l]}$and $\beta_{T[l]k2}^{[l]}$respectively, when the two haplotypes H*_k_*_1_ and H*_k_*_2_ carried by individual *k* fall into the two clusters. Thus, we can fit a logistic regression model to the data under the assumption of Hardy-Weinberg equilibrium and a multiplicative disease model:

$\eta_{k}=\log\left(\frac{p_{k}}{1-p_{k}}\right)=\beta_{T[l]k1}^{[l]}+\beta_{T[l]k2}^{[l]}+\sum_{c=1}^{C} \phi_{c}^{[l]}x_{kc}$ (A1)

where *p_k_* is the conditional probability of individual *k* being a case dependent on the two clusters containing two haplotypes, H*_k_*_1_ and H*_k_*_2_ carried by individual *k*. We can then obtain the log likelihood:

$L(y|T[l],\text{β}^{\text{[}l\text{]}},\phi^{[l]},\text{x})=\sum_{k=1}^{n} [y_{k}\ln p_{k}+(1-y_{k})\ln(1-p_{k})]$ (A2)

where *n* is the total sample size including case and control populations, and from Equation A1 *p_k_* can be rewritten as:

$p_{k}=\frac{\exp(\eta_{k})}{1+\exp(\eta_{k})}$ (A3)

Among all the partitions, T[1] can be regarded as the null partition, which means all *m* haplotypes share a single cluster. Hence the partition T[1] can denote the null hypothesis of no association between disease and haplotypes, i.e., all individuals including cases and controls have the equal odds of carrying each haplotype. Thus we construct an *LLR* to test disease-haplotype association for partition T[*l*]:

$LLR=-2[L(y|T[l],\text{β}^{\text{[}l\text{]}},\phi^{[l]},\text{x})-L(y|T[1],\text{β}^{\text{[1]}},\phi^{[1]},\text{x})]$ (A4)

Under the null hypothesis, statistic *LLR* in Equation A4 follows an approximate$\chi^{2}$distribution with *l*-1 df.

To allow for rare haplotypes, clusters with relative frequencies < 5% are pooled at partition T[*l*]. So the number of clusters in partition T[*l*] changes from *l* to *l**+1, and *l** denotes the number of clusters with relative frequencies ≥5%. After pooling the rare haplotypes at partition T[*l*], we have the new test statistic *LLR_pool_* which approximately follows a$\chi^{2}$distribution with *l** df.

For all partitions except T[1], we separately perform association analysis between disease and clusters of haplotypes based on an *LLR* test. From the bottom to the top of the cladogram, the number of clusters in partitions becomes gradually smaller until it equals 1, which results in a trade-off between reduced df in the *LLR* and lack of fitness for the logistic-regression model.

If there are *t_b_* partitions besides the T[1] partition included in a cladogram, we designate the best partition (T[best]) as the partition with the smallest *p-*value (*i.e.*, maximal *LLR* value) among *t_b_* separate *LLR* tests. T[best] shows the strongest evidence of disease/haplotype association. However we need to correct the raw *p-*values obtained in *t_b_* partitions for the additional levels of multiple testing. In our study, to obtain an experiment-wise false positive error rate of 0.05, the point-wise significance level is adjusted by Bonferroni correction 0.05/ *t_b_*.
